# Internet-Based Photoaging Within Australian Pharmacies to Promote Smoking Cessation: Randomized Controlled Trial

**DOI:** 10.2196/jmir.2337

**Published:** 2013-03-26

**Authors:** Oksana Burford, Moyez Jiwa, Owen Carter, Richard Parsons, Delia Hendrie

**Affiliations:** ^1^Curtin Health Innovation Research InstituteSchool of PharmacyCurtin UniversityPerthAustralia; ^2^Curtin Health Innovation Research InstituteCurtin UniversityPerthAustralia; ^3^Office of the Pro-Vice Chancellor (Health Advancement)Edith Cowan UniversityPerthAustralia; ^4^Curtin Health Innovation Research InstituteSchool of Pharmacy, Occupational Therapy and Social WorkCurtin UniversityPerthAustralia; ^5^Curtin Health Innovation Research InstituteCentre for Population Health ResearchCurtin UniversityPerthAustralia

**Keywords:** smoking, tobacco use disorder, skin aging

## Abstract

**Background:**

Tobacco smoking leads to death or disability and a drain on national resources. The literature suggests that cigarette smoking continues to be a major modifiable risk factor for a variety of diseases and that smokers aged 18-30 years are relatively resistant to antismoking messages due to their widely held belief that they will not be lifelong smokers.

**Objective:**

To conduct a randomized controlled trial (RCT) of a computer-generated photoaging intervention to promote smoking cessation among young adult smokers within a community pharmacy setting.

**Methods:**

A trial was designed with 80% power based on the effect size observed in a published pilot study; 160 subjects were recruited (80 allocated to the control group and 80 to the intervention group) from 8 metropolitan community pharmacies located around Perth city center in Western Australia. All participants received standardized smoking cessation advice. The intervention group participants were also digitally photoaged by using the Internet-based APRIL Face Aging software so they could preview images of themselves as a lifelong smoker and as a nonsmoker. Due to the nature of the intervention, the participants and researcher could not be blinded to the study. The main outcome measure was quit attempts at 6-month follow-up, both self-reported and biochemically validated through testing for carbon monoxide (CO), and nicotine dependence assessed via the Fagerström scale.

**Results:**

At 6-month follow-up, 5 of 80 control group participants (6.3%) suggested they had quit smoking, but only 1 of 80 control group participants (1.3%) consented to, and was confirmed by, CO validation. In the intervention group, 22 of 80 participants (27.5%) reported quitting, with 11 of 80 participants (13.8%) confirmed by CO testing. This difference in biochemically confirmed quit attempts was statistically significant (χ^2^_1_=9.0, *P*=.003). A repeated measures analysis suggested the average intervention group smoking dependence score had also significantly dropped compared to control participants (*P*<.001). These differences remained statistically significant after adjustment for small differences in gender distribution and nicotine dependence between the groups. The mean cost of implementing the intervention was estimated at AU $5.79 per participant. The incremental cost-effectiveness ratio was AU $46 per additional quitter. The mean cost that participants indicated they were willing to pay for the digital aging service was AU $20.25 (SD 15.32).

**Conclusions:**

Demonstrating the detrimental effects on facial physical appearance by using a computer-generated simulation may be both effective and cost-effective at persuading young adult smokers to quit.

**Trial Registration:**

Australian New Zealand Clinical Trials Registry: ACTRN12609000885291; https://www.anzctr.org.au/Trial/Registration/TrialReview.aspx?ACTRN=12609000885291 (Archived by WebCite at http://www.webcitation.org/6F2kMt3kC)

## Introduction

Tobacco smoking leads to premature death or morbidity and places a drain on national resources. Consequently, health professionals and governments stress the importance of smoking cessation and a reduction in exposure to tobacco smoking [1,2].

The younger people are when they start smoking, the greater the risk of illness or death caused by smoking [3]. Approximately half of smokers die prematurely from their habit, with half of these in middle age [4]. Smoking reduces life expectancy by approximately 7 years, with significant morbidity in the final years of a shortened life [4,5]. Even those who smoke between 1 and 4 cigarettes per day triple their long-term risk of dying from cardiovascular disease or lung cancer [6].

Currently in Australia, 19.7% of males and 16.3% of females aged 20 to 29 years smoke on a daily basis [7]. The detrimental long-term health effects of smoking, such as cardiovascular diseases and a variety of cancers, are generally well known in Australia [8]. However, health promotion research shows that, in isolation, knowledge about the hazards of smoking is insufficient to deter smoking behaviors [9]. Young adults who smoke are generally not concerned about the long-term health consequences of smoking because they may believe they will give up the habit while still young [10]. A number of previous studies have investigated the potential of personalized, computer-generated, facial aging software to prompt quit attempts in young adult smokers. These have found facial aging interventions to have some impact [11-14].

The objectives of this randomized controlled trial (RCT) were to test the efficacy and cost-effectiveness of an intervention based on personalized, vivid illustrations of “smoker’s face” among young smokers (18-30 years of age). Smokers face includes wrinkling of the face, gauntness of facial features, and a gray and plethoric complexion. Efficacy was assessed by comparing successful quitting, number of quit attempts, and change in smoking dependence (assessed by the Fagerström score) between the intervention and control groups. The study also aimed to explore the value (feasibility and cost) of an unfunded intervention within pharmaceutical practices.

## Methods

### Study Design and Population

This study was a RCT (Trial ID number: ACTRN12609000885291) that recruited 160 participants (80 participants assigned to both control and intervention groups) from 8 metropolitan community pharmacies located geographically around Perth city center, Western Australia, when presenting to collect prescribed medications or over-the-counter (OTC) medications.

Eligibility criteria included (1) age range of 18-30 years old (self-report); (2) smoker (defined as smoking 1 or more cigarettes per day via self-report); (3) able to give consent; (4) available for follow-up at 6 months; (5) no beards, mustaches, or non-removable facial accessories; (6) no body dysmorphia (participants screened using the Body Dysmorphic Disorder Questionnaire [BDDQ)]) [15]; and (7) not using nicotine replacement therapy (NRT) or taking oral drugs for nicotine dependence.

### Sample Size and Strategy

The sample size of 80 participants per group was calculated to observe a medium effect size (*d*=0.5), with 80% power and a type I error probability of 5%, and allowing for a 50% attrition rate. The anticipated effect size and attrition rates were based upon the results of a pilot study [16]. At each pharmacy, participants were recruited and assigned by the researcher to the different arms of the study on alternate weeks to minimize contamination between intervention and control participants. The study aimed to recruit 10 participants from each of the 8 pharmacies to each treatment arm (intervention or control). This stratification by pharmacy was performed in an attempt to avoid any bias due to socioeconomic factors.

### The Intervention

**Figure 1:**
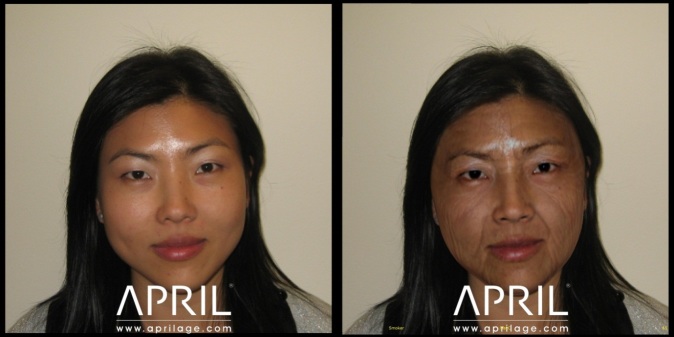
Current age photo (25 years) and future digitally aged photo (65 years) of a female current smoker.

**Figure 2:**
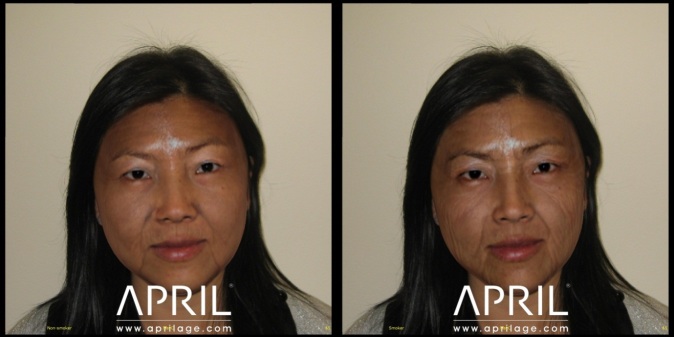
Digitally aged photos of female participant at 65 years as a nonsmoker (left) and as a smoker (right).

The APRIL Face Aging software is an Internet-based 3-dimensional age progression software package that creates a stream of aged images of faces from a standard digital photograph (the wrinkling/aging algorithms based upon normative data from people of a broad variety of ages, ethnicities, lifestyle habits, as well as published data regarding facial changes associated with aging). Additionally, the resulting aged images can be adjusted to compare how a person will age as a smoker versus as a nonsmoker (Figures 1 and 2).

### Data Collection

At recruitment, all participants were asked to complete a baseline questionnaire consisting of demographic data, the Fagerström Smoking Dependence Scale (score from 0-10) [17], questions concerning attitudes toward personal appearance, opinions about health risks associated with smoking, and perceived barriers to quitting smoking. Participants were recruited only if they were not using NRT and not taking oral drugs for nicotine dependence. Participants in both the intervention and control groups received standard 2-minute smoking cessation advice from the pharmacist.

**Figure 3:**
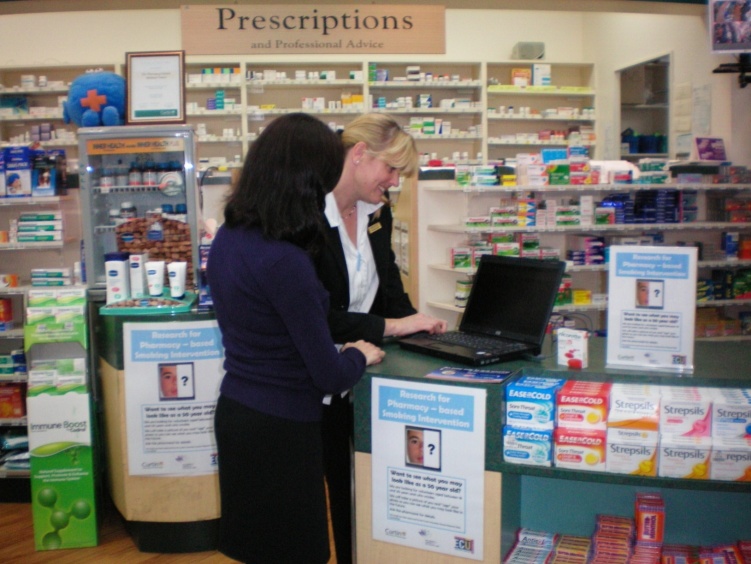
Intervention being delivered by pharmacist.

Participants in the intervention group were also screened for body dysmorphia using the BDDQ. In addition, they were photographed and their images were digitally aged as both a smoker and a nonsmoker (using the Internet-based APRIL Face Aging software), and invited to view the age-processed images (Figure 3). They were also asked to complete a questionnaire about their willingness to pay (WTP) for the digital aging service. The digitally aged photograph was sent to their email address within 24 hours of the intervention. Follow-up surveys were undertaken via telephone at 1, 3, and 6 months, each taking approximately 3 minutes to complete.

If participants stated at the 6-month follow-up that they had quit smoking, they were required within 48 hours to undertake a carbon monoxide (CO) breath test to validate their nonsmoking status. The CO monitor utilized was a portable, battery-operated Smokerlyzer (Bedfont Scientific Ltd, Kent, England, UK) that provided a CO level reading in parts per million (ppm).

### Primary Outcomes Measured

The primary outcomes measured were (1) the effect of the intervention by using successful quitting, quit attempts, and progression along the transtheoretical stages of change model, and (2) nicotine dependence using the Fagerström scale. These were measured at baseline and at 1-, 3-, and 6-month follow-ups.

The demographic and baseline smoking habit profiles of the recruited participants were compared between groups using Fisher’s exact test and Pearson’s chi-square test for categorical variables, and the Student’s *t* test for continuous variables. The primary endpoints of the study at the 6-month follow-up were analyzed using chi-square tests to compare percentages of quitters in each group, or *t* tests to compare smoking dependence levels. Percentages of quitters in each group were compared as both self-reported values and as CO-validated values. A logistic regression model was used to analyze the percentage of quitters in the 2 groups after adjustment for any possible differences between groups on the basis of demographic or baseline data. A repeated measures analysis (random effects regression model) was used to identify any changes in the Fagerström dependence score over the entire course of the study using 1- and 3-month follow-up surveys in addition to baseline and 6-month data. Data were analyzed using SAS v9.2 software (SAS Institute, Inc, Cary, NC, USA) with *P*<.05 taken to indicate a statistically significant association.

### Secondary Outcomes Measured

The secondary outcomes measured were (1) the cost-effectiveness of the intervention from a health sector perspective in terms of the incremental cost per additional quitter and per additional lifetime quitter, and (2) the business viability of delivering the intervention in a community pharmacy. These were calculated at the conclusion of the study.

Two perspectives were adopted: a health sector perspective and the perspective of a community pharmacy on the assumption that the intervention was not government funded. The direct costs of providing the digital aging service over and above providing standard cessation advice were calculated based on the time taken to provide the service and the cost to a pharmacy of purchasing tokens to use the online software to photoage participants. The cost of a pharmacist’s time was valued based on a published recommended rate of pay in Western Australia [18] and tokens were costed based on market price [19]. Time taken that was protocol driven was excluded. Potential cost offsets from a reduction in health care costs of quitters were used to calculate net intervention costs. Cost offsets were based on the Quit Benefits Model, which is a tool developed in Australia to predict the difference in health care costs of smokers and nonsmokers for males and females by age group after 10 years follow-up [20]. This follow-up period was considered long enough to show the beneficial impact of quitting, but short enough to remain within the time frame of policy makers. Cost offsets were discounted at a rate of 3% as recommended by the US Panel on Cost-effectiveness in Health and Medicine [21]. All costs were expressed in 2011 Australian dollars. The cost of the tokens was converted from American dollars to Australian dollars based on the average exchange rate in 2011 [22]. The number of lifetime quitters was calculated assuming a long-term smoking relapse rate of 37% within 10 years [23]. Smoking relapse after 10 years of abstinence has been found to be less than 1% per year [24].

To assess the robustness of the study results, a scenario sensitivity analysis with the best-case and worst-case scenarios were performed [25]. The parameters varied were the pharmacist’s time spent providing the service, the exchange rate for converting the cost of tokens from American dollars to Australian dollars, and the discount rate (Table 1).

In the best-case scenario, the pharmacist’s time was adjusted down by 25%, the exchange rate for converting American dollars to Australian dollars was varied to the lowest level in the past 5 years, and a discount rate of 0% was used [22]. In the worst-case scenario, the pharmacist’s time was adjusted up by 25%, the exchange rate was varied to the highest level in the past 5 years, and a discount rate of 5% was used. The quantitative data from the customer survey (WTP questionnaire) were analyzed using SPSS v17 software (SPSS Inc, Chicago, IL, USA). Customers’ perceptions about the value of the intervention and its impact on loyalty intentions and potential future sales were analyzed by using simple descriptive statistics.

**Table 1:** Parameter values for base case and sensitivity analyses.

Item^a^	Base case	Scenario sensitivity analysis	
		Best case	Worst case
Pharmacist time per participant to deliver service (mins)	4.8	3.6	6.0
Award wage rate per week for a pharmacist (AU$)	907.40	-	-
Cost of a token (AU$)	3.87	3.63	6.53
Exchange rate (AU$)	0.9687	0.9067	1.6321
Discount rate (%)	3	0	5

^a^ Compared to US $1.

## Results

### Study Design and Population

**Figure 4:**
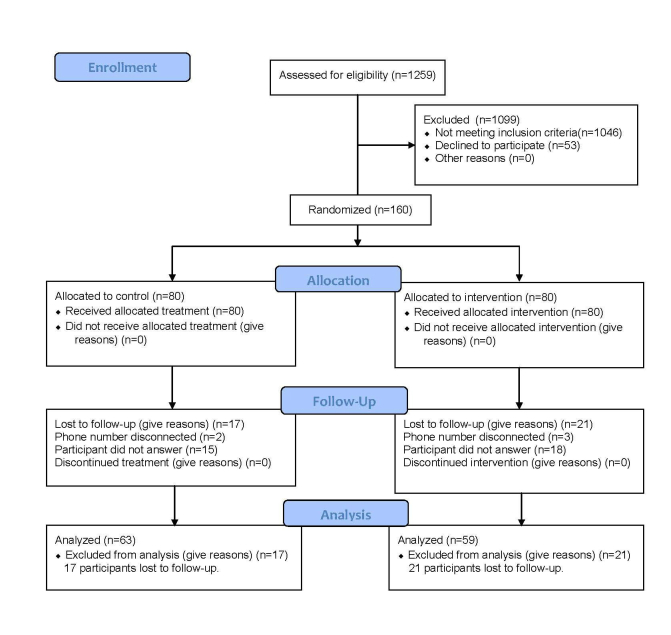
Profile of the randomized controlled trial (using CONSORT guidelines).

Customers were screened for eligibility to the RCT from 8 community pharmacies (Figure 4).

### Sample Size and Strategy

In total, 1259 customers were screened for eligibility; 213 customers were eligible and 160 were recruited, the others declined the invitation to participate for a range of very different reasons. Eighty participants were recruited to the control group and 80 to the intervention group.

### The Intervention

The smoker’s face simulations were created using a digital photograph (6.0 megapixels) taken of the intervention participants and uploaded to APRIL Face Aging software version 2.5 on a laptop computer.

### Data Collection

The RCT was conducted between January 2010 and December 2010 and all follow-up surveys were completed by June 2011. The final 6-month follow-up showed a response rate of 77.5% for the control group and 72.5% for the intervention group. The demographic and baseline smoking behaviors of recruited participants are shown and compared between groups (intervention versus control) in Table 2.

There were more females and lighter smokers (smoking up to 5 cigarettes per day) in the intervention group; however, there were no statistically significant differences between the control and intervention groups on demographic or smoking dependence variables at baseline. No participants were revealed to have body dysmorphia. A number of questions on the survey were designed to gather the respondents’ opinions of self-perceptions and their attitudes toward their smoking behavior. These questions were taken from an earlier survey [11], and showed that the groups were generally well matched. However, a greater proportion of the intervention group appeared to be concerned about their physical appearance (82.5% versus 67.5%, χ^2^_1_=4.8, *P*=.03), and believed that facial wrinkles were associated with smoking (98.8% versus 85.0%, χ^2^_1_=10.1, *P*=.002). There was no difference in the proportion of participants in each group who had made at least 1 attempt to quit smoking in the past (68.4% versus 70.9%, χ^2^_1_=0.1, *P*=.73).

**Table 2:** Demographic and baseline smoking profile of study participants (N=160).

Variable		Control group (n=80)	Treatment group (n=80)	*P* value^a^
**Gender, n (%)**				
	Male	35 (43.8)	25 (31.3)	.10
	Female	45 (56.2)	55 (68.7)	
Age, mean (SD)		25.1 (4.1)	24.2 (4.1)	.16^b^
**Education, n (%)**				.71
	Year 10 high school	15 (19.0)	17 (21.3)	
	Year 12 high school	31 (39.2)	29 (36.3)	
	Technical and further education (TAFE) qualifications	17 (21.5)	22 (27.5)	
	Degree (university/college)	16 (20.3)	12 (15.0)	
**Cigarettes per day over past 30 days, n (%)**				.35
	1	11 (13.8)	19 (23.8)	
	2-5	9 (11.3)	10 (12.5)	
	6-10	21 (26.3)	14 (17.5)	
	11-20	27 (33.8)	29 (36.3)	
	>21	12 (15.0)	8 (10.0)	
Fagerström score, mean (SD)		2.96 (2.52)	2.87 (2.48)	.82^b^
**Fagerström dependency score, n (%)**				.92
	0-2	39 (48.8)	39 (49.4)	
	3-4	19 (23.8)	18 (22.8)	
	5	8 (10.0)	10 (12.7)	
	6-7	10 (12.5)	10 (12.7)	
	8-10	4 (5.0)	2 (2.5)	

^a^ From chi-square test (unless otherwise marked) comparing the treatment groups.

^b^ From *t* test.

### Primary Outcomes Measured

Table 3 shows the response rates to the follow-up surveys, and the change in smoking behavior over the study duration. There was a significant difference in the proportion of participants self-reporting to have successfully quit smoking by the 6-month survey. Assuming that participants who failed to complete the final follow-up survey continued to smoke, only 1 of 80 control participants (1.3%, 95% CI 0-6.7) were confirmed nonsmokers compared to 11 of 80 participants in the intervention group (13.8%, 95% CI 7.8-22.9). This difference in confirmed quitting is statistically significant (χ^2^_1_=9.0, *P*=.003). The intervention group contained a larger proportion of participants responding to the question: “I care about how people think I look.” A logistic regression model was used to investigate the association between treatment group and self-reported quitting after adjustment for this difference as well as the small differences between groups in gender and nicotine dependence. The difference remained statistically significant after adjustment for these potentially confounding variables (*P*=.003).

A similar model using confirmed quitting as the dependent variable showed an adjusted *P* value for the treatment group of *P*=.03.

**Table 3:** Pattern of survey completion and change in smoking behavior at 6-month follow-up (N=160).

Variable		Control group (n=80)	Treatment group (n=80)	*P* value^a^
**Response to follow-up questionnaires, n (%)**				
	All surveys completed	56 (70.0)	48 (60.0)	.38
**Incomplete (last survey completed),** **n (%)**				
	6 month	6 (7.5)	10 (12.5)	
	3 month	8 (10.0)	14 (17.5)	
	1 month	3 (3.8)	4 (5.0)	
	No follow-up	7 (8.8)	4 (5.0)	
**Quit smoking at 6 months, n (%)**				
	Self-report (questionnaire)	5 (6.3)	22 (27.5)	<.001
	Confirmed (CO-validated)	1 (1.3)	11 (13.8)	.003
**Change in Fagerström smoking dependence score at 6 months, n (%)**				<.001^b^
	Reduced dependence	11 (13.8)	41 (51.3)	
	No change	68 (85.0)	39 (48.8)	
	Increased dependence	1 (1.3)	0	
**Change in mean Fagerström score from baseline**				<.001^c^
	At 1-month follow-up	–0.14	–0.83	
	At 3-month follow-up	–0.38	–1.34	
	At 6-month follow-up	–0.26	–1.88	

^a^ From chi-square tests unless otherwise specified.

^b^ From Fisher’s Exact test.

^c^ Obtained from a repeated measures analysis including all available surveys.

Table 3 also shows changes in the Fagerström smoking dependence score. The 6-month score was grouped into the 5 broad dependence level categories and compared with baseline data. There was a significant difference in change in smoking dependence between groups (χ^2^_2_=26.2, *P*<.001), with 14% of the control group moving to a lower category compared to 51% of the intervention group doing so.

A random effects regression model was used to model the mean change in Fagerström score from baseline by using data from all follow-up surveys The control group did not experience a significant drop in Fagerström score over the study (*P*=.36), whereas the participants in the intervention group dropped by an average of approximately 1.9 points (*P*=.002). The change in mean scores over the whole study was significantly different between treatment and control groups (*P*<.001).

Although there were no differences between participants at baseline, the regression models were extended to adjust for the gender and age of the participant, and the number of cigarettes smoked at baseline. The models were fitted to the control and intervention group separately because it was clear that changes in score appeared only in the intervention group. For the control group, there were no associations between change in score and age (*P*=.14), gender (*P*=.72), or baseline consumption (*P*=.49). However, for the intervention group, age (*P*<.001) and baseline consumption (*P*<.001) were significantly associated with change in score, whereas gender (*P*=0.34) was not associated. Older participants were less likely to reduce their score than younger participants (*P*=.001), suggesting that the intervention may have a greater effect on the younger participants. Participants who smoked more than 10 cigarettes per day showed a significant drop in score on the Fagerström scale of at least 1 point (*P*<.001) independent of age. Participants smoking 6 to 10 cigarettes per day obtained a lower score, but this change was not statistically significant (*P*=.07), whereas light smokers (0-5 cigarettes per day) showed no change in score.

### Secondary Outcomes Measured

Total costs of implementing the intervention from a health sector perspective were AU $463, or the equivalent of AU $5.79 per participant (Table 4).

**Table 4:** Economic analysis of photoaging service.

Item		Base case	Scenario sensitivity analysis	
			Best case	Worst case
Mean cost per participant of service (AU$)		5.79	5.07	8.93
Total cost of service^a^ (AU$)		463	406	714
**Incremental cost-effectiveness ratio (ICER)**				
	Cost per additional quitter (AU$)	46	41	71
	Cost per additional lifetime quitter (AU$)	74	64	113
Cost offset from reduction in health care costs (AU$)		2144	2660	1867
Net total cost savings (AU$)		1778	2346	1316
**Willingness to pay for service (AU$)**				
	Mean (SD)	20.25 (15.32)	-	-
	Median (IQR)	20.00 (10.00, 20.00)	-	-

^a^ For all 80 participants.

With an additional 10 quitters confirmed in the intervention group compared to the control group (11 and 1, respectively), the incremental cost-effectiveness ratio (ICER) was AU $46 per additional quitter, or the equivalent of AU $74 per additional lifetime quitter. Cost offsets of AU $2144 from a reduction in the health care costs of quitters resulted in the intervention potentially generating net total cost savings of AU $1778. In the best-case scenario, the ICER was AU $41 per additional quitter and net total cost savings were AU $2346. Corresponding figures for the worst-case scenario were AU $71 per additional quitter and AU $1316, respectively.

The mean cost that the participants indicated that they were willing to pay for the digital aging service was AU $20.25, which exceeded the mean cost per participant for delivering the service (AU $5.79). The median cost they were willing to pay was AU $20, similar to the mean value. More than 80% of participants said they would be more likely to use the pharmacy to purchase future smoking cessation therapies and to use it more for other purchases. More than 80% of participants also thought their friends would be willing to pay for the service, and all but 2 participants said they would recommend the photoaging intervention to 1 or more friends who were smokers.

## Discussion

### Summary of Findings

The impact of the photoaging innovation on confirmed quit attempts by the young people recruited to this study was statistically significant (*P*=.003). The data further demonstrate that the photoaging intervention had a larger influence on younger participants. Also, the participants who did not make a quit attempt, but who smoked more than 10 cigarettes per day, were likely to become less dependent on nicotine.

### Strengths and Weaknesses of the Study

The pharmacies selected to take part in the study were chosen to cover a range of socioeconomic areas and the equal number of study participants selected for each treatment group at each pharmacy aimed to diminish any potential biases. However, because of the nature of the intervention, the participants and researcher could not be blinded to the study group. Allocation to groups was not performed as eligible participants were recruited, but according to the treatment being used at the pharmacy during that week. In this setting, there was a substantial risk of contamination between treatment and control groups if participants had been randomized at the point of recruitment rather than by week of attendance at the pharmacy.

The baseline comparisons showed that the 2 groups were very similar on smoking dependence scores, and the 6-month follow-up response rate was high (over 70% for both groups). Follow-up to 12 months may have been preferable, but impractical, in this case. However, follow-up at 6 months was augmented by biochemical verification of tobacco use and cessation [26]. If participants stated they had made a quit attempt at the 6-month conclusion of the study, they were invited to undertake a CO monitor test to validate their nonsmoking status. It was disappointing that so few participants in the control group agreed to CO verification. There are 2 possible reasons for this: it is possible that they continued to smoke, or they were not as engaged in the project as the intervention group and were less amenable to follow-up. Nevertheless the self-reported smoking status data are interesting and although likely to be prone to socially desirable responses, the effect size is still substantial and on a par with other intervention trials.

Although there were more females and light smokers in the intervention group, this was not statistically significant and appeared not to diminish the significant statistical association between treatment group and quitting smoking.

### Strengths and Weaknesses in Relation to Other Studies

Although many individualized smoking cessation interventions have been implemented in the past few decades, few have had as marked an impact as reported here. With the advent of digital technology, quit messages can now be delivered by mobile telephone, email, text messaging, and online social networks [27].

To date, there have been few studies reporting on a personalized photoaging intervention [12-14]; those published have only recruited females, and only 1 of these studies was an RCT that recruited a small number of female smokers who had been referred to a smoking cessation service [14].

### Implications for Clinicians and Policy Makers

The economic analysis demonstrates that this personalized smoking cessation intervention is cheap and cost-effective, and it could be readily adopted in community pharmacies. It targets young smokers who are at significant risk of adverse effects of smoking if they continue lifelong smoking.

With an ICER of AU $74 per additional lifetime quitter, the intervention is cost-effective compared with other individualized smoking cessation programs. For example, a systematic review of economic evaluations of a range of smoking cessation interventions found ICERs of between US $260 to US $3263 per lifetime quitter (2002) for counseling or self-help programs versus usual care [28]. These ICERs are of a similar order of magnitude as reported elsewhere for brief advice from a general practitioner to quit smoking and smoking cessation counseling of £196 and £653 per lifetime quitter respectively (1999) [29]. Although these other studies calculated ICERs based on a societal perspective, additional non-health care costs of the photoaging interventions, such as patient time input, are minimal.

### Unanswered Questions and Future Research

A review commissioned by the Australian Commonwealth Department of Health and Ageing concluded that interventions delivered by health care providers significantly increased the number and success of quit attempts made in Australia each year [30].

Health care providers, such as pharmacists, are accessible and highly trained [31]. They have an established role in delivering smoking cessation pharmacotherapies and other forms of cessation assistance [32,33]. Could this intervention also be delivered by other health care providers in community settings, such as family medicine or allied health clinics?

A significant development for the use of mass media in delivering antismoking messages is the advent of digital technology. Technologies such as the Internet, social networking sites, and smartphones have the potential to reach large populations of younger people [2,28,34]. Could this Internet technology be delivered to the public without professional facilitation and would it have the same effect?

Further experimental research deploying photoaging technology is needed to answer these questions.

## Multimedia Appendix 1

CONSORT-EHEALTH checklist V1.6.2 [35].

## Abbreviations

BDDQ: Body Dysmorphic Disorder Questionnaire
CO: carbon monoxide
ICER: incremental cost-effectiveness ratio
NRT: nicotine replacement therapy
OTC: over-the-counter
RCT: randomized controlled trial
WTP: willingness to pay
