# Supplementary material for: Internet-Based Photoaging Within Australian Pharmacies to Promote Smoking Cessation: Randomized Controlled Trial
Source: J Med Internet Res. 2013 Mar 26;15(3):e64. doi: 10.2196/jmir.2337 (PMC3636310; doi:10.2196/jmir.2337)
Supplement: Supplementary file 1 [file jmir_v15i3e64_app1.pdf]

CONSORT-EHEALTH Checklist V1.6.2 Report

(based on CONSORT-EHEALTH V1.6), available at [http://tinyurl.com/consort-ehealth-v1-6].

2337

Date completed

1/31/2013 4:45:49

by

Burford

Internet based age progression simulation to promote smoking cessation facilitated in pharmacies in Australian primary care: A randomised controlled trial

TITLE

1a-i) Identify the mode of delivery in the title

Title is: " Internet based age progression simulation to promote smoking cessation facilitated in pharmacies in Australian primary care: A randomised controlled trial"

See also manuscript page 6 :

" Participants in both the intervention and control groups received standard two minute smoking cessation advice from the pharmacist. Participants in the intervention group were also screened for body dysmorphia using the BDDQ. In addition, they were photographed and their images digitally aged as a smoker and non-smoker (using the internet based April® Face Aging software), and invited to view the age-processed images."

1a-ii) Non-web-based components or important co-interventions in title

1a-iii) Primary condition or target group in the title

Title is: " Internet based age progression simulation to promote smoking cessation facilitated in pharmacies in Australian primary care: A randomised controlled trial"

Eligibility criteria included:
i)age range of 18–30 years old (self-report);
ii)smokers (defined as smoking one or more cigarettes per day - self-report);

ABSTRACT

1b-i) Key features/functionalities/components of the intervention and comparator in the METHODS section of the ABSTRACT

Abstract says: "All participants received standardised smoking cessation advice. Intervention group participants were also digitally 'photo-aged' by the researcher using internet based APRIL® Face Aging Software so they could preview images of themselves as a lifelong smoker and a non-smoker. "

1b-ii) Level of human involvement in the METHODS section of the ABSTRACT

1b-iii) Open vs. closed, web-based (self-assessment) vs. face-to-face assessments in the METHODS section of the ABSTRACT

1b-iv) RESULTS section in abstract must contain use data

1b-v) CONCLUSIONS/DISCUSSION in abstract for negative trials

INTRODUCTION

2a-i) Problem and the type of system/solution

See manuscript page 4:

" Young adults who smoke are generally not concerned about the long-term health consequences of smoking because they may believe they will give up the habit while still young [10]. A number of previous studies have investigated the potential of personalised, computer-generated, facial ageing software to prompt quit attempts in young adult smokers. These have generally found facial ageing interventions to have some impact [11-14].
The objectives of this randomised controlled trial were to test the efficacy and cost-effectiveness of an intervention based on personalised, vivid illustrations of 'smoker's face' amongst young smokers (18–30 years of age).....The study also aimed to explore the value (feasibility and cost) of an unfunded intervention within pharmacy practice."

2a-ii) Scientific background, rationale: What is known about the (type of) system

See manuscript page 4:

" The younger people are when they start smoking, the greater the risk of illness or death caused by smoking [3]. Approximately half of smokers die prematurely from their habit, with half of these in middle age [4]. Smoking reduces life expectancy by approximately seven years, with significant morbidity in the final years of a shortened life [4,5]. Even those who consume between one and four cigarettes per day triple their long-term risk of dying from cardiovascular disease or lung cancer [6].
Currently in Australia, 19.7% of males and 16.3% of females aged 20–29 years smoke on a daily basis [7]. The detrimental long-term health effects of smoking, such as cardiovascular diseases and a variety of cancers are generally well-known in Australia [8]. However, health promotion research shows that, in isolation, knowledge about the hazards of smoking is insufficient to deter smoking behaviours [9]. Young adults who smoke are generally not concerned about the long-term health consequences of smoking because they may believe they will give up the habit while still young [10]. "

METHODS

3a) CONSORT: Description of trial design (such as parallel, factorial) including allocation ratio

Page 4 of manuscript:

" The objectives of this randomised controlled trial were to test the efficacy and cost-effectiveness of an intervention based on personalised, vivid illustrations of 'smoker's face' amongst young smokers (18–30 years of age). Efficacy was assessed by comparing: successful quitting, number of quit attempts and change in smoking dependence (assessed by the Fagerström score), between the intervention and control groups. The study also aimed to explore the value (feasibility and cost) of an unfunded intervention within pharmacy practice."

3b) CONSORT: Important changes to methods after trial commencement (such as eligibility criteria), with reasons

No changes were introduced after trial commencement.

3b-i) Bug fixes, Downtimes, Content Changes

4a) CONSORT: Eligibility criteria for participants

" Eligibility criteria included:
i)age range of 18–30 years old (self-report);
ii)smokers (defined as smoking one or more cigarettes per day - self-report);
iii)able to give consent;
iv)available for follow-up at six months;
v)no beards, moustaches or non-removable facial accessories;
vi)no body dysmorphia; [participants screened using the Body Dysmorphic Disorder Questionnaire (BDDQ)] [15]
vii)not using nicotine replacement therapy (NRT) or taking oral drugs for nicotine dependence. "

4a-i) Computer / Internet literacy

4a-ii) Open vs. closed, web-based vs. face-to-face assessments:

Page 5 of manuscript:

"This study was a randomised controlled trial (RCT) [Trial ID number: ACTRN12609000885291] recruiting 160 participants (80 participants assigned equally to both control and intervention groups) from eight metropolitan community pharmacies geographically around Perth city centre, Western Australia, when presenting to collect prescribed medications or over the counter (OTC) medications."

Page 6:

" Participants in both the intervention and control groups received standard two minute smoking cessation advice from the pharmacist. "

4a-iii) Information giving during recruitment

4b) CONSORT: Settings and locations where the data were collected

"160 participants (80 participants assigned equally to both control and intervention groups) from eight metropolitan community pharmacies geographically around Perth city centre, Western Australia, when presenting to collect prescribed medications or over the counter (OTC) medications. "
Page 5

4b-i) Report if outcomes were (self-)assessed through online questionnaires

Page 6 of manuscript:

" At recruitment all participants were asked to complete a baseline questionnaire consisting of demographic data, the Fagerström Smoking Dependence Scale [score from 0–10] [17], questions concerning attitudes towards personal appearance, opinions about health risks associated with smoking, and perceived barriers to quitting smoking.
Participants in the intervention group were also screened for body dysmorphia using the BDDQ. In addition, they were photographed and their images digitally aged as a smoker and non-smoker (using the internet based April® Face Aging software), and invited to view the age-processed images. They were also asked to complete a questionnaire about their willingness to pay (WTP) for the digital ageing service.... Follow-up surveys were undertaken via telephone at one, three and six months and took approximately three minutes to complete.

At the six-month follow-up, if participants stated that they had quit smoking, they were reviewed within 48 hours to undertake a carbon monoxide (CO) breath test to validate their non-smoking status."

4b-ii) Report how institutional affiliations are displayed

5) CONSORT: Describe the interventions for each group with sufficient details to allow replication, including how and when they were actually administered

5-i) Mention names, credential, affiliations of the developers, sponsors, and owners

5-ii) Describe the history/development process

5-iii) Revisions and updating

5-iv) Quality assurance methods

5-v) Ensure replicability by publishing the source code, and/or providing screenshots/screen-capture video, and/or providing flowcharts of the algorithms used

5-vi) Digital preservation

5-vii) Access

Access to the platform and internet were as described on page 6:

" Participants were photographed and their images digitally aged as a smoker and non-smoker (using the internet based April® Face Aging software), and invited to view the age-processed images."

5-viii) Mode of delivery, features/functionalities/components of the intervention and comparator, and the theoretical framework

The internet based intervention was delivered by a pharmacist who also delivered the smoking cessation advice. The components of the intervention are described on page 5/6 :
" April® Face Aging Software is an internet based 3D age progression software package which creates a stream of aged images of faces from a standard digital photograph (the wrinkling/ageing algorithms based upon normative data from people of a broad variety of ages, ethnicities, lifestyle habits, as well as published data regarding facial changes associated with ageing). Additionally, the resulting aged images can be adjusted to compare how a person will age as a smoker versus a non-smoker."

5-ix) Describe use parameters

5-x) Clarify the level of human involvement

5-xi) Report any prompts/reminders used

Page 6 :
" The digitally-aged photograph was then sent to their nominated email address within 24 hours of the intervention. "

5-xii) Describe any co-interventions (incl. training/support)
A pharmacist delivered the following co-intervention: Page 2:

" All participants received standardised smoking cessation advice."

See also ANZCTR:

" Participants will receive 'one on one' standard smoking cessation advice by a pharmacist. This will comprise of them receiving a Pharmacy Self Care card on Smoking (a health information card from the Pharmacy Self Care Program of the Pharmaceutical Society of Australia) and verbal counselling from the pharmacist."

6a) CONSORT: Completely defined pre-specified primary and secondary outcome measures, including how and when they were assessed
Page 6:

" Primary outcomes measured:
i)the effect of the intervention using successful quitting, quit attempts, progression along the transtheoretical "stages of change" model;
ii)nicotine dependence using the Fagerström scale.
These were measured at the following stages: baseline, one, three and six months follow-up."

Page 7:
" Secondary outcomes measured:
i)the cost-effectiveness of the intervention from a health sector perspective in terms of the incremental cost per additional quitter and per additional lifetime quitter;
ii)the business viability of delivering the intervention in a community pharmacy.
These were calculated at the conclusion of the study."

6a-i) Online questionnaires: describe if they were validated for online use and apply CHERRIES items to describe how the questionnaires were designed/deployed

6a-ii) Describe whether and how "use" (including intensity of use/dosage) was defined/measured/monitored

6a-iii) Describe whether, how, and when qualitative feedback from participants was obtained

6b) CONSORT: Any changes to trial outcomes after the trial commenced, with reasons
No changes after trial commenced.

7a) CONSORT: How sample size was determined

7a-i) Describe whether and how expected attrition was taken into account when calculating the sample size

7b) CONSORT: When applicable, explanation of any interim analyses and stopping guidelines
Not applicable.

8a) CONSORT: Method used to generate the random allocation sequence
Page 5: " At each pharmacy participants were recruited and assigned by the researcher to the different arms of the study on alternate weeks to minimise contamination between intervention and control participants. The study aimed to recruit 10 participants from each of the eight pharmacies to each treatment arm (intervention or control). This stratification by pharmacy was performed in an attempt to avoid any bias due to socioeconomic factors."

8b) CONSORT: Type of randomisation; details of any restriction (such as blocking and block size)
Page 5: " At each pharmacy participants were recruited and assigned by the researcher to the different arms of the study on alternate weeks to minimise contamination between intervention and control participants. The study aimed to recruit 10 participants from each of the eight pharmacies to each treatment arm (intervention or control). This stratification by pharmacy was performed in an attempt to avoid any bias due to socioeconomic factors."

9) CONSORT: Mechanism used to implement the random allocation sequence (such as sequentially numbered containers), describing any steps taken to conceal the sequence until interventions were assigned
Page 5: " At each pharmacy participants were recruited and assigned by the researcher to the different arms of the study on alternate weeks to minimise contamination between intervention and control participants. The study aimed to recruit 10 participants from each of the eight pharmacies to each treatment arm (intervention or control). This stratification by pharmacy was performed in an attempt to avoid any bias due to socioeconomic factors."

10) CONSORT: Who generated the random allocation sequence, who enrolled participants, and who assigned participants to interventions
Page 5: " At each pharmacy participants were recruited and assigned by the researcher to the different arms of the study on alternate weeks to minimise contamination between intervention and control participants. The study aimed to recruit 10 participants from each of the eight pharmacies to each treatment arm (intervention or control). This stratification by pharmacy was performed in an attempt to avoid any bias due to socioeconomic factors."

11a) CONSORT: Blinding - If done, who was blinded after assignment to interventions (for example, participants, care providers, those assessing outcomes) and how

11a-i) Specify who was blinded, and who wasn't
Page 14/ 15: " ...due to the nature of the intervention the participants and researcher could not be blinded to the study group. Allocation to groups was not performed as eligible participants were recruited, but according to the treatment being used at the pharmacy during that week. In this setting there was a substantial risk of contamination between treatment and control group if participants had been randomised at the point of recruitment rather than by week of attendance at the pharmacy."

11a-ii) Discuss e.g., whether participants knew which intervention was the "intervention of interest" and which one was the "comparator"

11b) CONSORT: If relevant, description of the similarity of interventions
Not relevant

12a) CONSORT: Statistical methods used to compare groups for primary and secondary outcomes
This was not a cluster RCT as the intervention was facilitated by the same pharmacist.

12a-i) Imputation techniques to deal with attrition / missing values

LOCF' numbers are outlined in Table 3. (Incomplete: Last survey complete) e.g. at 6 months 8% of control group did not complete the follow up survey. Therefore these control group participants were assumed to continue as smokers or non smokers as last observed.

Page 11:

"Assuming that participants who failed to complete the final follow-up survey continued to smoke..."

12b) CONSORT: Methods for additional analyses, such as subgroup analyses and adjusted analyses

Page 11: " A logistic regression model was used to investigate the association between treatment group and self-reported quitting, after adjustment for this difference as well as the small differences between groups in gender and nicotine dependence. The P-value associated with the treatment group remained strongly significant after adjustment for these potentially confounding variables (P =.003). "

Page 13:
" Although there were no differences between participants at baseline, the regression models were extended to adjust for the gender and age of the participant, and the number of cigarettes smoked at baseline. The models were fitted to the control and intervention group separately, as it was clear that changes in score appeared only in the intervention group. For the control group, there were no associations between change in score and age (P =.14), sex (P =.72) or baseline consumption (P =.49). However, for the intervention group, age (P <.001) and baseline consumption (P <.001) were significantly associated with the change in score while gender (P =0.34) was not associated. Older participants were less likely to reduce their score than younger participants, suggesting that the intervention may have a greater effect on the younger participants. Participants who smoked more than 10 cigarettes per day showed a significant drop in score of at least one point on the Fagerström scale (P <.001), independently of age. Participants smoking 6–10 cigarettes per day showed a trend towards a lowering in score (P =.07), while light smokers (0–5 cigarettes per day) showed no change in score."

RESULTS

13a) CONSORT: For each group, the numbers of participants who were randomly assigned, received intended treatment, and were analysed for the primary outcome

Number of care providers =1. Care centres =8.

Pg 10: " In total, 1259 customers were screened for eligibility; 213 customers were eligible and 160 were recruited. Eighty participants were recruited to the control group and 80 to the intervention group."

13b) CONSORT: For each group, losses and exclusions after randomisation, together with reasons

See Figure 1. Page 9 manuscript.

13b-i) Attrition diagram

14a) CONSORT: Dates defining the periods of recruitment and follow-up

Page 10:
" The RCT was conducted between January 2010 and December 2010 and all follow-up surveys were completed by June 2011."

14a-i) Indicate if critical “secular events” fell into the study period

14b) CONSORT: Why the trial ended or was stopped (early)

Not applicable.

15) CONSORT: A table showing baseline demographic and clinical characteristics for each group

Page 10. Table 2.

15-i) Report demographics associated with digital divide issues

Page 10. Table 2.

16a) CONSORT: For each group, number of participants (denominator) included in each analysis and whether the analysis was by original assigned groups

16-i) Report multiple “denominators” and provide definitions

Figure 1 page 9.

16-ii) Primary analysis should be intent-to-treat

17a) CONSORT: For each primary and secondary outcome, results for each group, and the estimated effect size and its precision (such as 95% confidence interval)

Page 11:

" Assuming that participants who failed to complete the final follow-up survey continued to smoke, only one out of 80 control participants (1.3%, 95% Confidence Intervals 0% to 6.7%) were confirmed non-smokers, compared to 11 out of 80 participants (13.8%, 95% Confidence Intervals 7.8% to 22.9%) of the intervention group. "

Secondary outcomes; Table 4, page 13:

Mean willingness to pay for service (AUD) (SD)20.25 (15.32)
Median willingness to pay for service (AUD) [IQR]20.00 [10.00; 20.00]

17a-i) Presentation of process outcomes such as metrics of use and intensity of use

17b) CONSORT: For binary outcomes, presentation of both absolute and relative effect sizes is recommended

Not applicable.

18) CONSORT: Results of any other analyses performed, including subgroup analyses and adjusted analyses, distinguishing pre-specified from exploratory

Page 15 "We also noted a trend towards more females and light smokers in the intervention group, although on analysis this appeared not to diminish the very significant statistical association between treatment group and quitting smoking."

18-i) Subgroup analysis of comparing only users

19) CONSORT: All important harms or unintended effects in each group

Not applicable.

19-i) Include privacy breaches, technical problems

19-ii) Include qualitative feedback from participants or observations from staff/researchers

DISCUSSION

20) CONSORT: Trial limitations, addressing sources of potential bias, imprecision, multiplicity of analyses

20-i) Typical limitations in ehealth trials

Page 14-15:

" ... due to the nature of the intervention the participants and researcher could not be blinded to the study group. Allocation to groups was not performed as eligible participants were recruited, but according to the treatment being used at the pharmacy during that week. In this setting there was a substantial risk of contamination between treatment and control group if participants had been randomised at the point of recruitment rather than by week of attendance at the pharmacy.

The baseline comparisons showed that the two groups were very similar on smoking dependence scores, and the six-month follow-up response rate was high (over 70% for both groups). Follow-up to 12 months may have been preferable but impractical in this case. However follow-up at six months was augmented by biochemical verification of tobacco use and cessation [26]. If participants stated they had made a quit attempt at the six-month conclusion of the study, they were invited to undertake a CO monitor test to validate their non-smoking status. It was disappointing that so few participants in the control group agreed to CO verification. There are two possible reasons for this, it is possible that they continued to smoke or they were not as engaged in the project as the intervention group and were less amenable to follow-up. Nevertheless the self-reported smoking status data are interesting and although quite likely to be prone to socially desirable responses, the effect size is still substantial and on a par with other intervention trials. "

21) CONSORT: Generalisability (external validity, applicability) of the trial findings

21-i) Generalizability to other populations

21-ii) Discuss if there were elements in the RCT that would be different in a routine application setting

22) CONSORT: Interpretation consistent with results, balancing benefits and harms, and considering other relevant evidence

22-i) Restate study questions and summarize the answers suggested by the data, starting with primary outcomes and process outcomes (use)

This item is not applicable the the following reasons:

1. The intervention was facilitated by only one care provider. No cluster effect.

2. Blinding was not possible due to the nature of the intervention.

3. Control group could not be offered a similar intervention to the photoageing for smoking cessation, because other such interventions(e.g. to demonstrate obesity) are not comparable (i.e. this intervention does not lend itself to a sham equivalent). Photoageing for other substance abuse habits are not available.

22-ii) Highlight unanswered new questions, suggest future research

Other information

23) CONSORT: Registration number and name of trial registry

Trial ID Number: Australian New Zealand Clinical Trials Registry ACTRN12609000885291 http://www.anzctr.org.au/

24) CONSORT: Where the full trial protocol can be accessed, if available

Australian New Zealand Clinical Trials Registry ACTRN12609000885291 http://www.anzctr.org.au/

25) CONSORT: Sources of funding and other support (such as supply of drugs), role of funders

Page 17: This research was unfunded.

X26-i) Comment on ethics committee approval

x26-ii) Outline informed consent procedures

X26-iii) Safety and security procedures

X27-i) State the relation of the study team towards the system being evaluated
